# An Evaluation of Social Media and Geospatial Dating Apps for Recruitment of a National Sample of Young Men Who Have Sex with Men in the LITE-2 Study

**DOI:** 10.1007/s10461-026-05083-9

**Published:** 2026-02-27

**Authors:** Isabella Bonnewit, Rebecca Schnall, Robert Garofalo, Dustin T. Duncan, Olivia R. Wood, Michael Almodovar, Fengdi Xiao, Lisa M. Kuhns

**Affiliations:** 1 Division of Adolescent & Young Adult Medicine, Ann & Robert H. Lurie Children’s Hospital of Chicago, Chicago, USA; 2 School of Nursing, Columbia University, New York, USA; 3 Department of Epidemiology, Columbia University, Mailman School of Public Health, New York, USA

**Keywords:** HIV prevention, Recruitment, Dating app, Sexual and gender minorities

## Abstract

In 2022, over two thirds of individuals diagnosed with HIV in the United States were people of color, half resided in the south, and 67% of new cases were attributed to male-to male sexual transmission (Centers For Disease Control, 2024). To combat these health disparities, the Ending the HIV Epidemic (EHE) initiative recommends that HIV prevention research focus on targeted populations and geographic regions with high rates of new HIV diagnosis. There are limited data on the relative efficiency of social media and dating apps for the recruitment of key EHE populations to HIV prevention studies. The LITE-2 Study aimed to recruit a national sample of approximately 3,000 young men who have sex with men (YMSM), at least 30% Black/African American and 30% Hispanic/Latino. This analysis compared the success of different social media platforms in relation to study goals, using descriptive statistics from the LITE-2 study (N = 2999) to assess enrollment count, eligibility rates, cost, and geographic distribution for each platform. Facebook had the highest enrollment rate, 45.38%, among eligible screeners. The cost per enrolled participant for Grindr, Sniffies, Scruff, Jack’d, and Adam4Adam was $118.02, $129.46, $220.59, $252.53, and $305.56 respectively. Jack’d had the highest proportion of Black participants, and Sniffies the highest proportion of Hispanic/Latino individuals. Use of Scruff resulted in enrollment of the highest proportion of participants in the rural EHE jurisdictions (47.1%). These findings inform digital recruitment strategies for future studies with similar racial/ethnic and geographic targets.

## Introduction

The Ending the HIV Epidemic in the US (EHE) initiative seeks to reduce new HIV infections by 75% by 2025 and 90% by 2030, in part by focusing on populations whose rates of new diagnoses are highest [1]. Target populations in the US include men who have sex with men (MSM), who accounted for 67% of new diagnoses in 2022, Black and Latino individuals (70% of new diagnoses), people age 13-34 (56% of new diagnoses) and individuals living in rural areas, especially the South (52% of new diagnoses in 2020) [1]. Social determinants of health, such as poverty, racism, and homophobia have a significant impact on HIV transmission in the United States (U.S.). These impacts are experienced differently in urban and rural areas of the U.S. [2].

These minoritized groups are disproportionally represented in HIV research. Black and Latino individuals are less likely to participate in research due to a myriad of historical and modern barriers [3]. By recruiting participants who are highly susceptible to HIV transmission, the LITE-2 study will estimate HIV incidence in young men who have sex with men (YMSM) over time, as well as related factors for HIV transmission, which will inform targeted interventions [4].

To reach members of these key populations from across the nation, the use of internet-based recruitment for clinical research has become commonplace [5]. Online venues such as social media and dating apps are especially useful for HIV-related studies because minority populations, who are disproportionately impacted by HIV, use social media at increased rates than non-minority populations [6, 7]. Conversely, online recruitment modes present unique challenges to obtain a study sample that represents *all* groups affected by HIV, such as individuals living in rural areas [7–9]. Therefore, it is imperative that research studies employ targeted online outreach strategies to (1) reach total enrollment goals, (2) recruit efficiently and within budget, and (3) represent EHE priority populations.

The literature offers some insight into the relative success and costs of different modes of recruitment for HIV prevention studies. Evidence is limited to the most popular platforms such as Facebook, Instagram, and Twitter, whereas newer platforms like TikTok, which are widely used by youth, are not well studied [7]. Facebook is a commonly used and cost-effective social media platform for study recruitment [10]. The median cost per enrolled participant across 35 health related survey-based studies was $14.41 [5]. Facebook survey respondents have shown to be representative of the general population in terms of drug use and STI rate, but whiteness is somewhat overrepresented [5, 11]. When the target audience is adolescents (age 13–18), however, enrollment was 8.4 times larger on Instagram [12]. Facebook shares its parent company, Meta, with Instagram, so ads can be connected between the two social medias, which is more cost effective [10]. Evidence suggests that Twitter is an ineffective recruitment tool in terms of enrollment, making it an expensive and less common recruitment mode [10].

Geosocial dating apps are another mode of recruitment for HIV prevention studies. Gay dating apps are an important potential source of recruitment because they reach people who are sexually active [13] and are accessible to otherwise non-disclosed MSM [14]. There is limited literature on the success of HIV-related study recruitment on traditionally heterosexual dating apps such as Tinder or Hinge. Among LGBTQ+ men who have ever used a dating app, 42% have used Tinder, 13% have used Hinge, and 62% have used Grindr, an app targeted for gay, bisexual, and queer men [16]. Therefore, many HIV-related studies utilize gay dating apps to recruit participants [15]. In an HIV prevention study in New York City, NY, and San Juan, Puerto Rico, the recruitment sources yielding the highest rates of eligibility from screening were Grindr (32.65%, n = 16 were eligible), Jack’d (29.41%, n = 15), and Growlr (28.89%, n= 13) [17]. In a separate HIV prevention study focused on racial minority MSM and involving at-home HIV tests, (n = 125, 70.6%) participants were recruited from Jack’d, and (n = 9, 5.0%) from Grindr [18]. Dating apps were the most cost-effective recruitment method at an average cost of $88.80 per enrolled participant, compared to $491.60 for social media (Facebook and Instagram) recruitment for a study involving at-home HIV testing [18]. A survey-based study on gay dating app use revealed that Black MSM frequently use Jack’d (32.6%) and Adam4Adam (30.3%) significantly more than white men [19].

The LITE-2 study recruited a national cohort of over 3,000 young men who have sex with men (YMSM), trans-male and non-binary individuals age 17–29. The study enrolled and followed this large sample for 24 months, with visits every 6 months to assess HIV incidence and related factors. The LITE-2 study had several recruitment goals. First, the study aimed to enroll at least 30% participants who identify as Black/African American (non-Hispanic). Second, the study aimed to recruit 30% participants who identify as Hispanic/Latinx (all races). Thirdly, LITE-2 aimed to recruit 50% of participants from EHE-priority jurisdictions and 50% of participants outside of these jurisdictions [4].

Herein, we evaluate the recruitment in the LITE-2 study by analyzing the efficiency and cost of each recruitment mode (Grindr, Sniffies, Jack'd, Scruff, Adam4Adam, and Facebook/Instagram) in reaching target groups for enrollment by race, ethnicity, geographic representation, and age.

## Methods

### Recruitment Procedure

This paper focuses on study participants who were enrolled in the LITE-2 study via online advertisements from October 2022 through October 2024 [4]. The advertisements featured images of YMSM, primarily YMSM of color, as part of our strategy to intentionally seek out this population. Advertisements were not standardized across platforms, but were updated regularly to reflect changing trends that could impact interest in the study. Interested individuals were prompted by the advertisement to click on a link to access the online screener, which is located in REDCap software (REDCap 13.1.35). Duplicate screeners were removed from the analysis during weekly quality control checks. In order to be eligible to participate in the LITE-2 study, participants are required to be (1) between the ages of 17 and 29, (2) identify as male, trans male, or non-binary, (3) understand English and/or Spanish, (4) live in the US or US territories, (5) self-report having had anal sex in the past 12 months with someone who has a penis, and (6) self-report HIV-negative status, status unknown, or diagnosed HIV positive in the past 12 months. Study staff reached out via text to individuals who screened eligible to set up a baseline visit, which was completed via video conference or asynchronously. The baseline visit included photo ID verification, and the completion of a survey and an at-home (OraQuick) HIV test [4]. Enrolled participants were compensated with a $45 electronic gift card at the baseline visit and were eligible to receive $365 over the course of the longitudinal study.

### Recruitment Modes

An advertising company was hired to execute advertising campaigns on dating apps, including Grindr, Adam4Adam, Sniffies, Scruff, Facebook and Jack’d. These advertisements included a link to the screening form, which assessed an individual’s eligibility for participation in LITE-2.

### Study Outcomes

To examine the efficiency and efficacy of each recruitment mode, recruitment data were analyzed cross sectionally at the conclusion of the recruitment period. The recruitment mode was embedded in a link source code for each campaign. Participant link source was not recorded for the first five months of recruitment. This dataset includes both participants who were recruited during those months (labeled “No Link Source”) and participants for whom a link source was recorded for the remainder of the study period. Outcomes were calculated by comparing metrics between recruitment modes including total enrollment, total eligible, total screened, and corresponding eligible/screened and enrolled/eligible ratios. Eligibility ratios were determined by dividing the number of eligible screeners by the number of eligible and ineligible screeners. Cost per enrolled participant was calculated by dividing the cost of advertising on a given platform by the total number of enrolled participants with the screener link source associated with that platform. Race was measured by the multiple-select survey question, “Do you consider yourself: (Check all that apply).” Participants who selected multiple races, and those who self-selected the “multiracial” answer option, were categorized as “multiracial.” Ethnicity was measured by the survey question, “Are you Hispanic/Latino/Latinx?” Participants who identified as Hispanic/Latino/Latinx, regardless of their racial selection, were categorized as Hispanic/Latino/Latinx. Both race and ethnicity questions were optional. Participants who did not provide an answer to either question were categorized as “Unknown.” Geographic distribution was analyzed by asking, “What is the zip code of your permanent address?” Age was calculated at the time of enrollment based on the participant’s date of birth. Participants who did not meet the age criteria at the enrollment visit (17-29) were removed from this analysis (n = 2).

Given the present study’s focus on describing recruitment yields and the demographic characteristics of participants reached through different digital platforms, all analyses are descriptive in nature. We did not conduct inferential analysis because recruitment was non-random and platform audiences differ in their demographics and behavioral patterns.

The LITE-2 study was approved by the Institutional Review Board at Columbia University, which served as the single IRB of Record. The IRB waived documentation of consent for the collection of contact information for screening. All participants who enrolled completed written informed consent in the baseline visit. Unique ID numbers were assigned to screeners and enrolled participants to ensure privacy.

## Results

For this analysis, we include 13,421 individuals who had complete screening data and 2,999 of 3,227 enrolled participants with complete recruitment source data for analysis (Table 1). We conducted approximately 24 total ad campaigns on Grindr, 6 ad campaigns on Adam4Adam, 4 campaigns on Scruff, 6 on Jack’d, and 6 on Sniffies. We conducted ongoing intermittent advertisements on Facebook/Instagram. Grindr produced the most cases in terms of total enrollment (n = 1,881). The mode of recruitment with the highest conversion of enrollment among those who were eligible was Facebook at 45.38% (n=59 enrollees/n=130 eligible screeners). Next, 23.99% of eligible screeners from Scruff enrolled in the LITE-2 study, followed by Jack’d (20.82%), Sniffies (20.22%), Grindr (19.93%), and Adam4Adam (14.66%). The most cost-effective recruitment mode was Grindr, with a cost of $118.02 per enrolled participant. This was followed by Sniffies, with an advertising cost of $129.46 per enrolled participant. The average cost per enrolled participant on Scruff was $220.59 and Jack’d was $252.53 per enrollee. Recruitment on Adam4Adam was the most expensive, at $305.56 per enrollee.

**Table 1:** Enrollment eligibility rate and cost by recruitment source

Recruitment source	*n* eligible	Enrolled/eligible rate	*n* enrolled	Cost per enrolled participant
Grindr	9,439	19.93%	1881	$118.02
Sniffies	1,108	20.22%	224	$129.46
Jack’d	951	20.82%	198	$252.53
Scruff	567	23.99%	136	$220.59
Adam4Adam	614	14.66%	90	$305.56
Facebook and Instagram	130	45.38%	59	*
Total	13,421	22.35%	2,999	

^*^Cost calculation Facebook is excluded due to missing data

^**^n = 411 missing Link source data

The racial and ethnic demographics by link source are reported in Table 2. This includes participants with and without a recorded link source and complete race/ethnicity and EHE data (n = 2,993). We were able to recruit an overall racially and ethnically diverse sample, with only (n = 1,223, 40.9%) identifying as White/Caucasian and non-Hispanic/Latino. However, the study did not meet its goal to recruit 30% of participants who identify as Black/African American. Among enrolled participants, 12.8% (n = 384) identified as Black/African American. The study did meet the 30% Hispanic/Latino goal, with n=884 (29.5%) of participants self-identifying as Hispanic/Latino. Individuals identifying as Asian make up 6.2% of the sample (n = 187), individuals identifying as multiracial make up 5.9% of the sample (n= 178), and individuals identifying as American Indian, Alaska Native, Native Hawaiian or other Pacific Islander make up 0.4% of the sample (n = 12). 4.2% of participants did not respond to race or ethnicity questions or selected “Something else” (n = 125).

**Table 2:** Enrolled cases by race and recruitment source (N = 2993)

Recruitment source	White	Hispanic/Latino/Latinx, all races	Black/African American	Asian	Multiracial	Native Hawaiian, Pacific Islander, American Indian or Alaskan Native	Unknown or something else	Total
Grindr *n (%)*	816 (43.5)	556 (29.6)	197 (10.5)	104 (5.5)	106 (5.6)	9 (0.5)	90 (4.7)	1878
Sniffies	72 (32.1)	86 (38.4)	26 (11.6)	17 (7.6)	13 (5.8)	–	10 (0.9)	224
Jack’d	62 (31.6)	37 (18.9)	61 (31.1)	10 (5.1)	14 (7.1)	1 (0.5)	11 (5.6)	196
Scruff	54 (39.7)	33 (24.3)	30 (22.1)	7 (5.1)	9 (6.6)	–	3 (2.2)	136
Adam4Adam	28 (31.1)	22 (24.4)	24 (26.7)	6 (6.7)	4 (4.4)	–	6 (6.7)	90
Facebook and Instagram	29 (49.2)	19 (32.2)	2 (3.4)	7 (11.9)	1 (1.7)	–	1 (1.7)	59
No Link Source	162 (39.5)	131 (32.0)	44 (10.7)	36 (8.8)	31 (7.6)	2 (0.4)	4 (1.0)	410
	1223 (40.9)	884 (29.5)	384 (12.8)	187 (6.2)	178 (5.9)	12 (0.4)	125 (4.2)	2993

^*^n=6 missing race

The app that led to recruitment of the highest proportion of enrolled participants who identified as Black/African American was Jack’d (n = 61, 31.1%). Grindr sourced the largest total enrollment of individuals identifying as Black/African American (n = 197, 10.5% of total recruited from Grindr). Sniffies yielded the highest proportion of participants identifying as Hispanic/Latino (n = 86, 38.4%). The app that resulted in enrollment of the largest total number of Hispanic/Latino participants was also Grindr (n = 556, 29.6%).

Geographic EHE Jurisdiction for enrolled participants by recruitment source is reported in Table 3 (n = 2,993). Nearly half, 44.9% (n = 1343), of enrolled participants to the LITE-2 study reside in an EHE priority jurisdiction (Table 3). In terms of recruitment mode, study recruitment on Grindr resulted in the highest total number of enrollees living in a priority jurisdiction (n = 813, 43.3%). Greater than half of enrollees from Adam4Adam (n = 53, 58.9%) and Scruff (n = 72, 53.0%) live in EHE priority jurisdictions. The EHE priority jurisdictions were less represented among Jack’d (n = 96, 49.0%), Sniffies (48.1%, n = 110), and Facebook enrollees (n = 25, 42.4%).

**Table 3:** Geographic EHE jurisdiction (County, State) for enrolled participants by recruitment source (N=2993)

Recruitment source	Priority county n (%)	Priority state n (%)	Non-EHE jurisdiction n (%)	Total
Grindr	688 (36.7)	125 (6.7)	1064 (56.7)	1877
Sniffies	99 (44.2)	11 (4.9)	114 (50.9)	224
Jack’d	82 (41.8)	14 (7.1)	100 (51.0)	196
Scruff	64 (47.1)	8 (5.9)	64 (47.0)	136
Adam4Adam	40 (44.4)	13 (14.4)	37 (41.1)	90
Facebook and Instagram	24 (40.7)	1 (1.7)	34 (57.6)	59
No link source	144	30	237	411
Total	1141	202	1650 (55.1)	2993

^*^n=6 missing EHE data

**Fig. 1:**
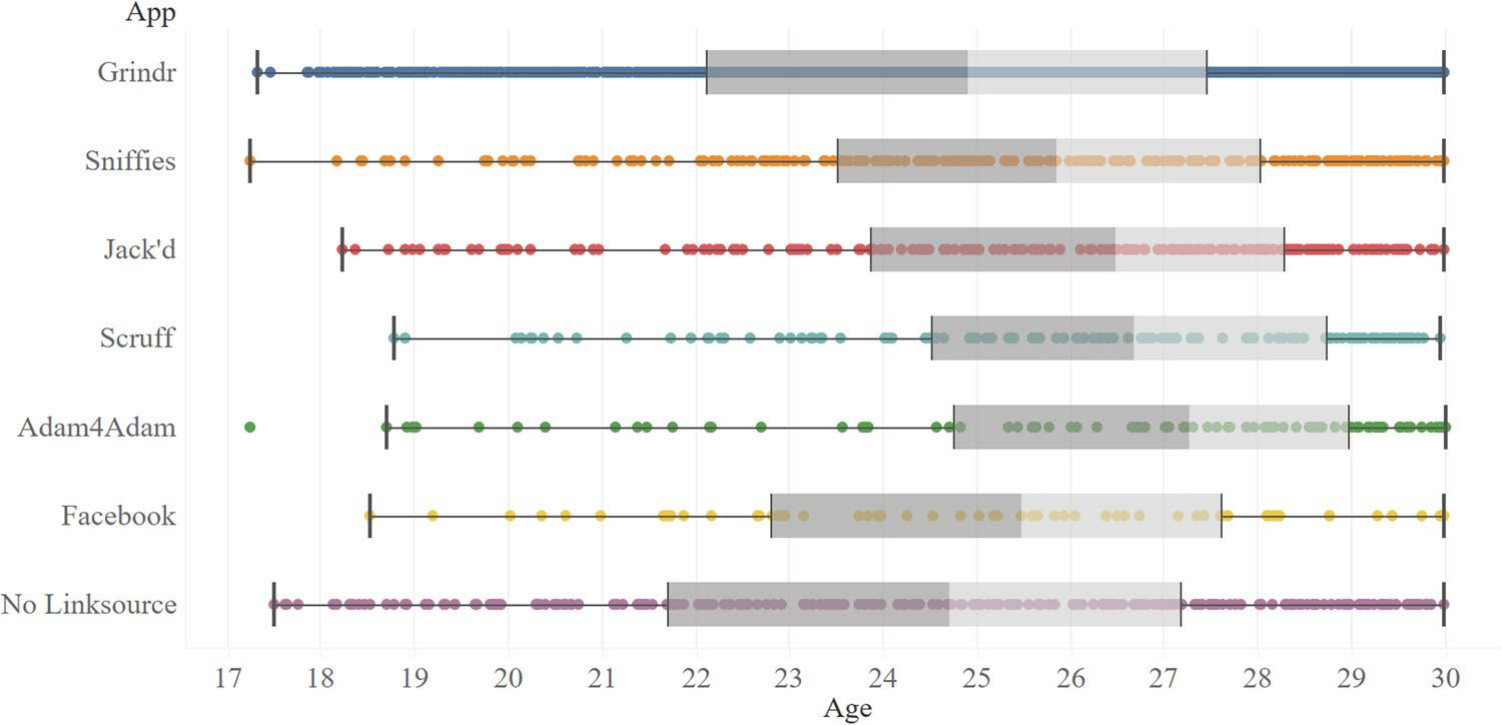
Age by recruitment source (N = 2991)

Among enrolled participants with available age data (n = 2,817), the mean age is 25.38 and the median age is 25.72 (SD = 3.11), and the distribution is skewed to the left (Figure 1). The minimum age is 17.25 and the maximum age is 29.99. Participants recruited via Adam4Adam had the highest median age at 27.27 years old (SD = 3.29). Scruff enrolled participants had a median age of 26.68 (SD = 2.87), followed by Jack’d at 26.49 (SD = 2.98), Sniffies at 25.85 (SD = 3.02), and Facebook at 25.48 (SD = 2.97). Grindr was the recruitment source with the youngest overall median age at 24.90 (SD = 3.09).

## Discussion

The recruitment of a large and racially diverse sample in alignment with the EHE priority populations is essential for accurate data collection in nationwide HIV research studies. The LITE-2 study, with its national scope and large sample size, offers insight about future recruitment from gay dating apps. This paper describes the relative success of each dating app in meeting the LITE-2 recruitment goals of a large sample, racial demographics (30% Hispanic/Latino and 30% Black/African American), and geographic distribution (50% enrollees living in an EHE priority jurisdiction).

Consistent with previous literature, our study confirms that geospatial network-based dating app recruitment reaches more people of color compared to social media, but a low proportion of Black/African American individuals specifically [18]. The existing recruitment techniques have been relatively successful in reaching the 50% EHE jurisdiction target, with 44.9% of enrollees living in EHE-designated counties and states. Although there are no specific age targets for this study, and the age distribution was met (17-29), the sample is skewed toward older participants, with only 6.5% of the sample under 20 years old. This study provides preliminary information about the recruitment capabilities of newer, non-Grindr gay dating apps (Adam4Adam, Sniffies, Scruff and Jack’d), which were similar to Grindr in terms of mean age of enrolled participants, but without the absolute reach of Grindr in terms of numbers enrolled.

Grindr is an important mode of recruitment due to its sheer number of users [16]. Considering its immense popularity, the LITE-2 study allotted the highest advertising budget for Grindr. Compared to the other gay dating apps, Grindr boasts the highest rates of eligibility, and the highest total enrollment. Among enrolled participants with available recruitment data, 62.7% (n = 1,881) were recruited from Grindr. Analysis revealed that Grindr recruitment yielded the lowest cost per enrolled participant compared to the other dating apps at $118.02 per enrollee. Unfortunately, the low proportion (10.5%) of Black/African American enrollment from Grindr contributes to the low overall proportion of Black/African American-identifying participants in the LITE-2 study. This is consistent with a 2021 study in which Grindr was the recruitment mode that yielded the second-most eligible participants, but only 10.7% (n = 28) identified as Black/African American [20]. Future studies should employ robust advertising on Grindr but consider how advertisement design could better appeal to Black/African American users, such as increasing imagery of Black/African American MSM [21]. Qualitative research also suggests that to engage and retain Black/African American MSM, there should be study staff that identifies as Black/African American MSM, and that cultural competency is important for retention [22]. There is limited research, however, on online recruitment strategies that specifically target Black/African American YMSM.

Smaller gay dating apps have slightly different membership demographics than Grindr, and may offer unique recruitment opportunities. There is very limited existing literature on the use of Sniffies, an app launched in 2018, as a research recruitment source. A high proportion (n = 86, 38.4%) of individuals recruited by Sniffies identified as Hispanic/Latino, while (n = 26, 11.6%) identified as Black/African American. Sniffies was on par with Grindr in terms of cost per enrolled participant at $129.46. The data indicates that Sniffies is a cost-effective option for recruiting Hispanic/Latino YMSM.

Scruff, likewise, yielded a relatively high enrolled/eligible rate (23.99%) of Hispanic/Latino participants. Given that Scruff was among the most costly platforms per enrolled participant, Sniffies may represent a more cost-effective option between the two.

The Jack’d advertisement campaign was the second-most expensive at $252.53 per enrolled participant. Jack’d drew in the highest proportion of Black/African American enrollees (31.1%, n = 61). Conversely, it drew the lowest proportion of enrollees identifying as Hispanic/Latino (18.9%). This supports earlier findings that identified Jack’d a valuable recruitment tool for YMSM of color [18].

Adam4Adam recruited a relatively low number of enrolled participants to LITE-2 (n = 90). Compared to the other platforms, Adam4Adam recruited the highest proportion of enrollees living in an EHE-designated state (14.4%, n = 13), suggesting it may have potential for recruitment in rural areas. Its low eligibility rate among those screened (8.69%) and high cost per enrolled participant ($305.56), however, demonstrates its low efficacy as a recruitment tool for the LITE-2 study.

Facebook and Instagram ads demonstrated potential to reach participants of younger age groups, which are underrepresented in the enrolled LITE-2 sample. A key disadvantage of Facebook/Instagram recruitment is its tendency to enroll a high proportion (51.67%) of white participants, confirming previous findings [20]. Although our study is missing the data for the cost per enrolled participant from this platform, it should be noted that a 2021 study with a comparable number of enrolled participants from Facebook/Instagram (n = 109) spent $155.09 per participant [20]. Facebook and Instagram advertisements should be increased in the future to reach the full age spectrum, but strategies to target these ads toward people of color should be considered.

Our findings confirm that dating app recruitment approaches can be leveraged to reach populations for whom traditional recruitment approaches are insufficient. For example, qualitative research shows that rural YMSM strongly value anonymity and prefer digital methods of outreach compared to traditional media or in-person recruitment [23, 24]. The present study demonstrates that dating apps were an effective approach for recruiting participants from rural EHE-designated states (n = 202).

Previous studies have used social media to recruit adolescent YMSM, but few have recruited via dating app advertisements [25]. Despite in-app restrictions, about 10–19% of adolescents under 18 use online dating sites [26, 27]. Sexual minority youth have significantly greater odds of online dating compared to their heterosexual peers [26]. The present study shows preliminary evidence for the successful recruitment of sexually active gay and bisexual adolescents aged 18 on various gay dating apps.

The dating app recruitment approach is particularly advantageous for research involving sexually active populations, offering access to diverse and geographically dispersed participants. Future research involving different study populations or study designs may also benefit from leveraging dating app advertisements to expand reach and improve recruitment efficiency.

Overall, this project provides information about the research recruitment capabilities of newer, non-Grindr gay dating apps (Adam4Adam, Sniffies, Scruff and Jack’d) and Facebook/Instagram. Use of these apps for recruitment largely led to the success of the LITE-2 study to meet target geographic distribution goals, age distribution, and its goal of a study sample that is 30% Hispanic/Latino, but did not lead to achievement of the goal of 30% of participants identifying as Black/African American. Areas for future research include evaluating recruitment efficiency of YMSM across a wider range of digital platforms and apps. Additionally, future studies should address the intersecting, underlying factors that hinder research recruitment among Black/African American YMSM by developing targeted strategies to adequately reach this population.

### Limitations

One limitation is that the LITE-2 study did not collect data on the recruitment mode of screened and enrolled participants from the outset of data collection, so we may under or overestimate effectiveness by platform. In addition, the advertisement campaigns on the various social media platforms were not equal, with more campaigns on platforms with higher reach, such as Grindr and Jack’d, which impacts the comparison of absolute numbers. Platform-specific differences in user demographics may have influenced recruitment outcomes, as each platform attracts distinct groups of users. This study does not examine the different advertisements used and how ad design may have contributed to higher engagement of certain populations.
